# TIMM13 as a prognostic biomarker and associated with immune infiltration in skin cutaneous melanoma (SKCM)

**DOI:** 10.3389/fsurg.2022.990749

**Published:** 2022-08-17

**Authors:** Sitong Zhou, Yuanyuan Han, Ronghua Yang, Xiaobing Pi, Jiehua Li

**Affiliations:** ^1^ Department of Dermatology, The First People’s Hospital of Foshan, Foshan, China; ^2^ Institute of Medical Biology, Chinese Academy of Medical Sciences and Peking Union Medical College, Yunnan Key Laboratory of Vaccine Research and Development on Severe Infectious Diseases, Kunming, China; ^3^ Department of Burn and Plastic Surgery, Guangzhou First People's Hospital, South China University of Technology, Guangzhou, China

**Keywords:** cutaneous melanoma, translocase of inner mitochondrial membrane 13 (TIMM13), prognosis, biomarker, tumor immune microenvironment

## Abstract

**Objective:**

Providing protection against aggregation and guiding hydrophobic precursors through the mitochondria’s intermembrane space, this protein functions as a chaperone-like protein. SLC25A12 is imported by TIMM8 as a result of its interaction with TIMM13. In spite of this, it is still unknown how TIMM13 interacts with skin cutaneous melanoma (SKCM) and tumor-infiltrating lymphocytes (TILs).

**Methods:**

Aberrant expression of TIMM13 in SKCM and its clinical outcome was evaluated with the help of multiple databases, including the Xiantao tool (https://www.xiantao.love/), HPA, and UALCAN. TISIDB and Tumor Immune Estimation Resources (TIMER) databases were applied to explore the association between TIMM13 and tumor infiltration immune cells. OS nomogram was constructed, and model performance was examined. Finally, TIMM13 protein expression was validated by immunohistochemistry (IHC).

**Results:**

TIMM13 expression was higher in SKCM samples than in peritumor samples. TIMM13 was strongly associated with sample type, subgroup, cancer stage, lymph node stage, and worse survival. Further, upregulation of TIMM13 was significantly associated with immunoregulators, and chemokines, as well as T cells, B cells, monocytes, neutrophils, macrophages, and T-cell regulators. An analysis of bioinformatic data uncovered that TIMM13 expression was strongly associated with PD1 (T-cell exhaustion marker). The nomogram showed good predictive performance based on calibration plot. TIMM13 was highly expressed in melanoma tissue samples than in normal samples.

**Conclusion:**

In brief, TIMM13 may be a prognostic biomarker for SKCM. It might modulate the tumor immune microenvironment and lead to a poorer prognosis. In addition, it is necessary to study the targeted therapy of TIMM13.

## Introduction

There are two main types of melanoma: cutaneous and uveal (1). Melanin-producing melanocytes in the skin are transformed into melanoma cells through malignant transformation (2). More than 75% of skin cancer deaths are caused by melanoma, even though it represents only 5% of all skin cancer cases (3). Cutaneous melanoma is considered the most aggressive type of skin cancer (4). There are approximately 232,100 (1.7%) new cases of cutaneous melanoma diagnosed worldwide (excluding nonmelanoma skin cancers), and the incidence of cutaneous melanoma is around 55,500 deaths per year (0.7% of all cancer deaths) (5). Before the FDA’s approved targeted and immunotherapy strategies in 2011 and 2014, metastatic melanoma was thought to be incurable and not treatable (6). These strategies are effective in treating some cases of melanoma, but not all, due to toxicity, intrinsic resistance to drugs, and other unidentified causes (7). It is necessary to perform surgery, radiotherapy, chemotherapy, and conduct clinical trials to cure resistant melanoma cells (8). With SKCM, the discovery of new immunotherapy targets and the detection of immune-related biomarkers are urgent tasks.

The TIMM13 gene encodes a chaperone protein that is part of the evolutionarily conserved TIMM (translocase of inner mitochondrial membrane) family that assists in the transport of proteins into mitochondria from the cytosol (9). The TIMM13 gene prevents hydrophobic precursors from aggregating and guides the proteins passing the mitochondrial intermembrane. SLC25A12/ARALAR1 and SLC25A13/ARALAR2 are imported by the TIMM8-TIMM13 complex (10). Timm13 is associated with Mohr-Tranebjaerg Syndrome and Visual Cortex Disease (11). It is related to the Peroxisomal lipid metabolism pathway (12). Studies have shown that TIMM13 was differently expressed in metastatic susceptibility (13), hepatocellular carcinoma (14), breast cancer (15) et al. These research proposed that TIMM13 may impact cancer profoundly, and it may be considered a new target for dealing with various malignant tumors. However, the possible mechanisms of TIMM13 in tumorigenesis and immune involvement in SKCM is still unclear.

In the study, the expression of TIMM13 and its connection with the clinical outcomes of SKCM were explored by various databases, including the Xiantao tool, HPA, and UALCAN databases. In addition, the Xiantao tool and TIMER database was conducted to explore the connection of TIMM13 with the infiltration of immune cells. The TISIDB database was used to study the relationship between TIMM13 and immunomodulators, immunostimulators, and chemokines. This research revealed the important function of TIMM13 in SKCM, and then the possible relationship and possibility of TIMM13 regulating tumor invasion of immune cells.

## Materials and methods

### Interaction analysis of gene expression profiling

The Xiantao tool online database (https://www.xiantao.love/) (16) is an integrated platform using R software (3.6.3) for obtaining analytical data from TCGA (https://portal.gdc.cancer.gov/) (17) and The Genotype–Tissue Expression (GTEx) (https://gtexportal.org/home/) (18). R packages used in the Xiantao tool were set as default. In the study, we evaluated TIMM13 expression in SKCM and normal tissues using the Xiantao data source.

### UALCAN database analysis

The UALCAN website (http://ualcan.path.uab.edu/) contains massive bioinformatics and clinical data on 31 TCGA malignant tumors (19). It can be used to make a gene expression comparison between SKCM and normal samples, at different tumor stages, different subtypes, and other clinicopathological characteristics. In the research, the expression level of TIMM13 will be determined by the main clinicopathological characteristics, such as tissue category (healthy or tumor), SKCM stage (stages 1, 2, 3, 4), lymph node stage (N01 stage, 2, 3), cancer subgroup, etc. The clinicopathological features (tumor degree, stage) and TIMM13 expression distribution of patients were collected by the UALCAN database and Xiantao tool for correlation analysis.

### Survival analysis for SKCM

Based on the median of the expression of TIMM13, patients were divided into a high TIMM13 expression group and a low TIMM13 expression group. The prognostic significance of TIMM13 in SKCM, including overall survival (OS), and progression-free interval (PFI), were explored using the Xiantao tool between the high TIMM13 expression group and low TIMM13 expression group. The hazard ratio (HR) with 95% confidence intervals and log-rank p-value was estimated. *P* < 0.05 was regarded as statistical significance.

### Tumor immune infiltration analysis

ssSGSEA method was applied to explore the potential connection between TIMM13 mRNA expression and immune infiltration level in SKCM by TIMER 2.0 (http://timer.cistrome.org/) (20). In addition, TILs gene markers and TILs gene expression were analyzed by the Xiantao tool. Then we drew the expression dispersion maps between a pair of custom genes for SKCM and the statistical significance of the correlation was estimated by Spearman's method. log2 RSEM was applied to exhibit the levels of gene expression.

### TISIDB database analysis

TISIDB (http://cis.hku.hk/TISIDB/) (21) is an online platform for studying tumor-immune system interaction, which combines various heterogeneous data sources. This database can assist us to know the interaction between tumors and immune cells, predict the responses of the body to immunotherapy, and find out novel immunotherapy targets. It will be a precious basis for tumor immunology studies and treatment. In this study, we used TISIDB to study the relationship between TIMM13 and 28 TILs, 45 immunopotentiators, 24 immunosuppressants, 41 chemokines, and 18 receptors in SKCM.

### Building and validation of a nomogram

A nomogram is a statistical model of prognosis presented as a simple graph. In the nomogram, each sample is assigned a point for each of its variables and the resulting total score predicts 1-, 3-, and 5-year survival rates. We used independent prognostic factors to build a nomogram using the “rms” package. A calibration plot (by a bootstrap method with 500 resamples) was used to validate the nomogram and concordance index (C-index).

### Immunohistochemistry (IHC) staining

Paraffin-embedded tissues were sectioned at 4 μm for IHC analysis. Antigens were retrieved by incubating the samples in citrate buffer (pH 6.0) for 15 min at 100 °C in a microwave oven and naturally cooled to room temperature. After blocking with a mixture of methanol and 0.75% hydrogen peroxide, sections were incubated overnight with appropriate dilatation of primary antibodies (TIMM13, Invitrogen, 1:150) followed by incubation with a secondary antibody conjugated with HRP (goat anti-rabbit, 1:500, Cell Signalling Technology). The sections were washed three times with PBS and incubated with AEC (ZSGB-BIO).

### Statistical analysis

Statistical analyses were performed with the Student t test, the Mann-Whitney U test, or analysis of variance, as appropriate. For nonparametric (non-normally distributed) data, Mann–Whitney U test was used for statistical analysis. The survival curve was drawn by KM plot by log-rank test. We calculated Spearman's correlation coefficient to explore the relationship between TIMM13 expression and immune infiltration levels, immunomodulators, and chemokines. Statistically significant was regarded as *p* < 0.05.

## Results

### High expression of TIMM13 in SKCM tissue

**Figure 1:**
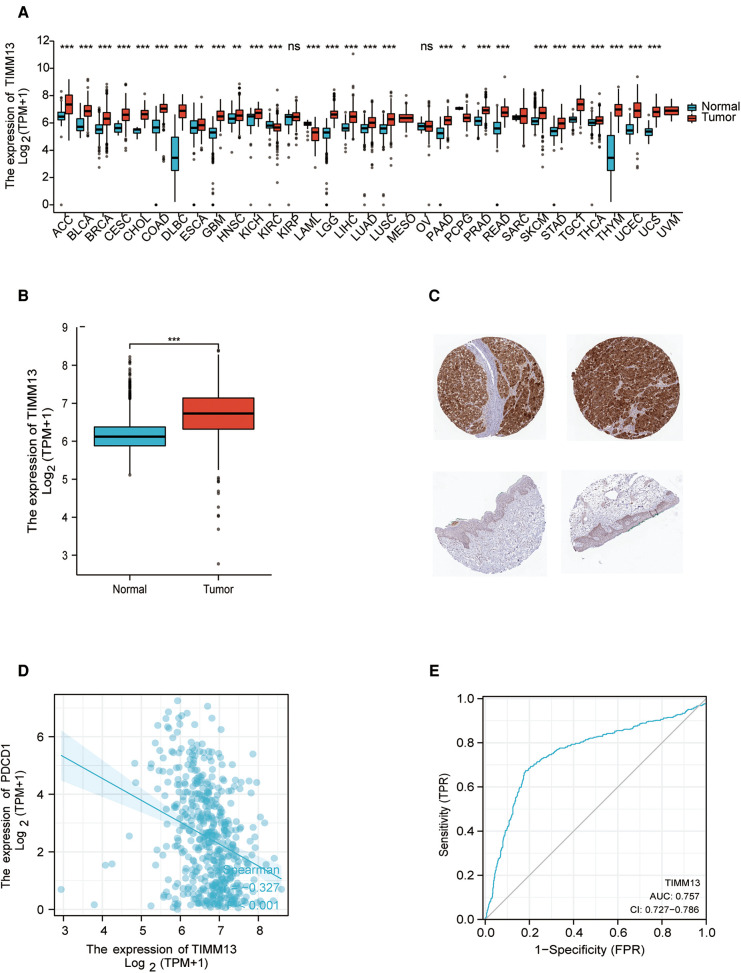
TIMM13 expression levels. (**A**) Increased or decreased TIMM13 in different tumor types from The Cancer Genome Atlas (TCGA) database were determined by Xiantao tool (*p < 0.05, **p < 0.01, ***p < 0.001). (**B**) Increased TIMM13 in SKCM tissues compared with normal tissues. (**C**) TIMM13 immunohistochemical staining levels in SKCM tissues (up) and normal tissues (down) by using HPA database. (**D**) The correlation analysis between TIMM13 and PD1 mRNA level. (**E**) The receiver-operating characteristic (ROC) curve analysis of TIMM13 in SKCM patients.

The differential expression of TIMM13 in tumor and normal tissues were studied by RNA-seq data of different types of cancer from TCGA. The conclusion was shown in Figure 1A. According to Xiantao tool, we found that the expression of TIMM13 upregulated in almost all cancer tissues such as ACC, BLCA and SKCM, but downregulated in KIRC, LAML and PCPG.

To validate these results in SKCM, cancer samples from the TCGA database and normal samples from GTEx database were tested by xiantao tool. As shown in Figure 1B, it was found that TIMM13 mRNA expression was significantly higher in SKCM samples (469 cases) than that in normal samples (813 cases) (*p* < 0.05) from GETx. Based on the HPA website, immunohistochemistry confirmed the TIMM13 protein expression pattern (Figure 1C). It is worth noting that the increased expression of TIMM13 mRNA was negatively correlated with PD1 (Figure 1D), where the AUC of TIMM13 in SKCM is 0.757 (95% CI is 0.727 to 0.786) (Figure 1E). It is suggested that TIMM13 was significantly elevated in SKCM tissues, which may be a potential biomarker for the diagnosis of SKCM.

### Relationship between TIMM13 expression and patient clinical pathology in SKCM

**Figure 2:**
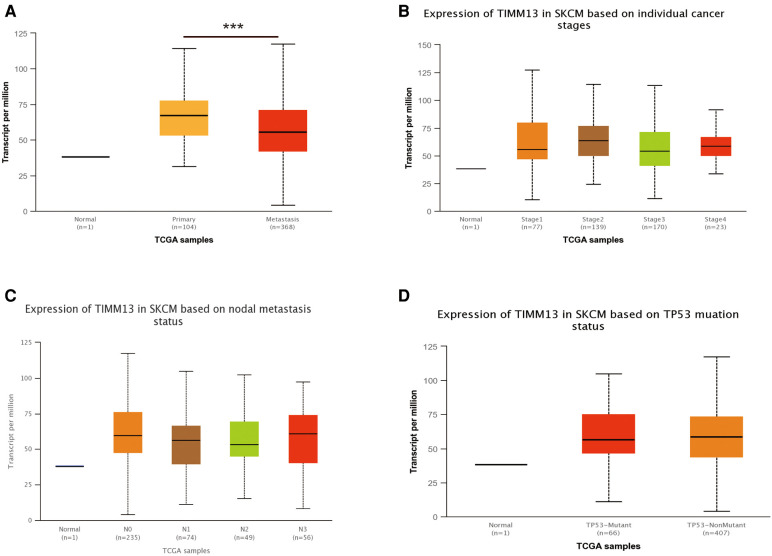
Correlation between TIMM13 mRNA expression level and clinicopathological parameters of SKCM through the UALCAN database. (**A**) Sample type (normal/primary tumor). (**B**) Cancer stage (stage 1, 2, 3, and 4). (**C**) Lymph node stage (N0 1, 2, and 3). (**D**) TP53 mutation subgroup. N, normal; P, primary tumor; S1, stage 1; S2, stage 2; S3, stage 3; S4, stage 4.

Using the UALCAN and the Xiantao tool, we investigated the relationship between TIMM13 expression and several clinicopathological features, such as tumor type, stage, lymph node stage, and TP53 mutation shown in Figure 2A, the expression of TIMM13 in SKCM metastatic cancer tissues was significantly higher than that in primary cancer tissues and normal cancer tissues (*p* = 0. 0018). There was no significant difference between different cancer stages (Figure 2B), different lymph nodes (Figure 2C) and TP53 mutation (Figure 2D). Secondly, in order to further understand the function and possible mechanism of TIMM13 in tumorigenesis, we used univariate and multivariate Cox regression analysis to observe the correlation between TIMM13 expression and clinicopathological features of SKCM. The up-regulation of TIMM13 expression was related to a poorer OS (HR = 2. 199, *p* < 0.001) (Table 1), DSS (Table 2) (HR = 2. 442, *p* < 0.001) and PFI (Table 3) (HR = 1. 439, *p* = 0. 027) in SKCM patients. The increase of TIMM13 mRNA expression was related to a poorer OS in T4 stage (HR = 4. 500, *p* = 0. 010), N2 stage (HR = 3. 617, *p* = 0. 048), N3 stage (HR = 11. 432, *p* < 0.001), melanoma ulceration (HR = 1. 650, *p* = 0. 030), and Melanoma Clark V level (HR = 4. 519, *p* = 0.043) SKCM patients. Furthermore, we found that DSS and PFI in T4 stage (DSS HR = 3.415, *p* = 0.039), N1 stage (DSS HR = 4.736, *p* = 0.044), N2 stage (DSS HR = 5.521, *p* = 0.027), N3 stage (DSS HR = 17.411, *p* < 0.001; PFI HR = 5.829, *p* < 0.001), Melanoma Clark V level (DSS HR = 5.338, *p* = 0.028; PFI HR = 3.211, *p* = 0.021) were associated with TIMM13 expression. It was a hint that the prognostic effect of TIMM13 in SKCM might be influenced by their clinical characteristics.

**Table 1:** Univariate and multivariate Cox regression analysis of OS.

Characteristics	Total (*N*)	Univariate analysis		Multivariate analysis	
		Hazard ratio (95% CI)	*p-*value	Hazard ratio (95% CI)	*p-*value
Gender	456				
Female	172	Reference			
Male	284	1.172 (0.879-1.563)	0.281		
Pathologic stage	410				
Stage I	77	Reference			
Stage II	140	1.586 (1.054-2.385)	**0.027**	0.279 (0.111-0.703)	**0**.**007**
Stage III	170	1.983 (1.344-2.927)	**<0**.**001**	0.166 (0.040-0.695)	**0**.**014**
Stage IV	23	3.517 (1.781-6.944)	**<0**.**001**	0.221 (0.047-1.045)	0.057
T stage	361				
T1	41	Reference			
T2	77	1.495 (0.811-2.756)	0.197	1.529 (0.620-3.768)	0.356
T3	90	2.097 (1.158-3.798)	**0**.**015**	2.924 (0.964-8.871)	0.058
T4	153	3.711 (2.070-6.653)	**<0**.**001**	4.500 (1.435-14.111)	**0**.**010**
N stage	402				
N0	224	Reference			
N1	73	1.497 (1.014-2.210)	**0**.**043**	3.489 (0.988-12.327)	0.052
N2	49	1.534 (0.972-2.419)	0.066	3.617 (1.012-12.930)	**0**.**048**
N3	56	2.731 (1.769-4.215)	**<0**.**001**	11.432 (3.201-40.822)	**<0**.**001**
M stage	430				
M0	406	Reference			
M1	24	1.897 (1.029-3.496)	**0**.**040**		
Tumor tissue site	392				
Extremities	190	Reference			
Trunk	166	0.940 (0.693-1.275)	0.691		
Head and Neck	36	1.269 (0.755-2.131)	0.368		
Melanoma ulceration	313				
No	146	Reference			
Yes	167	2.085 (1.495-2.907)	**<0**.**001**	1.650 (1.050-2.592)	**0**.**030**
Melanoma clark level	315				
I and II	19	Reference			
III	76	1.679 (0.704-4.002)	0.243	2.340 (0.606-9.039)	0.218
IV	167	2.945 (1.286-6.747)	**0**.**011**	2.704 (0.701-10.435)	0.149
V	53	5.449 (2.253-13.177)	**<0**.**001**	4.519 (1.046-19.532)	**0**.**043**
Age	456				
≤60	246	Reference			
>60	210	1.656 (1.251-2.192)	**<0**.**001**	1.258 (0.847-1.870)	0.256
TIMM13	456				
Low	230	Reference			
High	226	1.711 (1.307-2.241)	**<0**.**001**	2.199 (1.498-3.228)	**<0**.**001**

Bold values represent significantly different.

**Table 2:** Univariate and multivariate Cox regression analysis of DSS.

Characteristics	Total (*N*)	Univariate analysis		Multivariate analysis	
		Hazard ratio (95% CI)	*p*-value	Hazard ratio (95% CI)	*p-*value
Gender	450				
Female	172	Reference			
Male	278	1.161 (0.855-1.575)	0.340		
Pathologic stage	405				
Stage I	76	Reference			
Stage II	139	1.496 (0.977-2.291)	0.064	0.299 (0.115-0.773)	**0.013**
Stage III	167	1.777 (1.181-2.672)	**0**.**006**	0.125 (0.024-0.660)	**0**.**014**
Stage IV	23	3.800 (1.914-7.547)	**<0**.**001**	0.178 (0.031-1.019)	0.052
T stage	356				
T1	41	Reference			
T2	76	1.435 (0.775-2.657)	0.250	1.390 (0.557-3.470)	0.481
T3	88	1.934 (1.060-3.529)	**0**.**032**	2.562 (0.835-7.863)	0.100
T4	151	3.110 (1.715-5.637)	**<0**.**001**	3.415 (1.066-10.944)	**0**.**039**
N stage	396				
N0	221	Reference			
N1	71	1.279 (0.827-1.976)	0.269	4.736 (1.041-21.537)	**0**.**044**
N2	49	1.473 (0.905-2.398)	0.120	5.521 (1.212-25.162)	**0**.**027**
N3	55	2.920 (1.867-4.565)	**<0**.**001**	17.411 (3.852-78.698)	**<0**.**001**
M stage	424				
M0	400	Reference			
M1	24	2.200 (1.190-4.069)	**0**.**012**		
Tumor tissue site	386				
Extremities	188	Reference			
Trunk	164	0.920 (0.664-1.274)	0.615		
Head and neck	34	1.271 (0.720-2.244)	0.407		
Melanoma ulceration	309				
No	145	Reference			
Yes	164	1.948 (1.372-2.767)	**<0**.**001**	1.593 (0.993-2.555)	0.053
Melanoma clark level	310				
I and II	19	Reference			
III	76	1.898 (0.739-4.877)	0.183	2.270 (0.580-8.891)	0.239
IV	163	3.235 (1.307-8.005)	**0**.**011**	2.814 (0.723-10.951)	0.136
V	52	5.779 (2.206-15.141)	**<0**.**001**	5.338 (1.204-23.670)	**0**.**028**
Age	450				
≤60	244	Reference			
>60	206	1.699 (1.258-2.294)	**<0**.**001**	1.246 (0.819-1.895)	0.304
TIMM13	450				
Low	227	Reference			
High	223	1.798 (1.348-2.399)	**<0**.**001**	2.442 (1.621-3.677)	**<0**.**001**

Bold values represent significantly different.

**Table 3:** Univariate and multivariate Cox regression analysis of PFI.

Characteristics	Total (*N*)	Univariate analysis		Multivariate analysis	
		Hazard ratio (95% CI)	*p-*value	Hazard ratio (95% CI)	*p-*value
Gender	457				
Female	172	Reference			
Male	285	1.037 (0.821-1.309)	0.763		
Pathologic stage	411				
Stage I	77	Reference			
Stage II	140	1.381 (0.980-1.944)	0.065	0.411 (0.193-0.877)	**0.021**
Stage III	171	1.968 (1.426-2.715)	**<0**.**001**	0.363 (0.112-1.179)	0.092
Stage IV	23	3.438 (1.989-5.942)	**<0**.**001**	0.379 (0.115-1.254)	0.112
T stage	362				
T1	41	Reference			
T2	77	0.995 (0.639-1.548)	0.982	1.196 (0.598-2.390)	0.613
T3	91	1.325 (0.859-2.044)	0.203	1.817 (0.770-4.287)	0.173
T4	153	2.129 (1.387-3.268)	**<0**.**001**	1.975 (0.812-4.802)	0.133
N stage	403				
N0	224	Reference			
N1	74	1.634 (1.183-2.258)	**0**.**003**	2.496 (0.888-7.014)	0.083
N2	49	1.502 (1.023-2.205)	**0**.**038**	2.579 (0.904-7.353)	0.076
N3	56	2.965 (2.096-4.195)	**<0**.**001**	5.829 (2.132-15.933)	**<0**.**001**
M stage	431				
M0	407	Reference			
M1	24	2.026 (1.255-3.269)	**0**.**004**		
Tumor tissue site	393				
Extremities	190	Reference			
Trunk	167	1.075 (0.834-1.386)	0.577		
Head and neck	36	1.364 (0.866-2.150)	0.180		
Melanoma ulceration	313				
No	146	Reference			
Yes	167	1.626 (1.228-2.152)	**<0**.**001**	1.443 (0.982-2.121)	0.062
Melanoma clark level	315				
I and II	19	Reference			
III	76	1.333 (0.709-2.505)	0.372	1.170 (0.496-2.758)	0.720
IV	167	1.908 (1.048-3.473)	**0**.**034**	1.223 (0.513-2.915)	0.650
V	53	4.821 (2.486-9.349)	**<0**.**001**	3.211 (1.193-8.645)	**0**.**021**
Age	457				
≤60	247	Reference			
>60	210	1.576 (1.240-2.002)	**<0**.**001**	1.395 (0.988-1.970)	0.059
TIMM13	457				
Low	230	Reference			
High	227	1.303 (1.041-1.631)	**0**.**021**	1.439 (1.041-1.990)	**0**.**027**

Bold values represent significantly different.

### Increased TIMM13 mRNA expression associated with poor overall survival in SKCM patients

**Figure 3:**
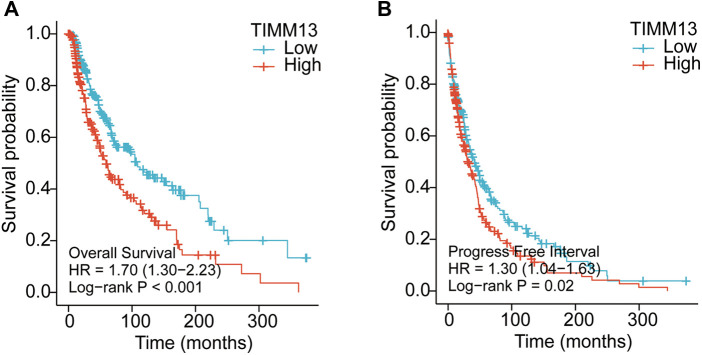
Kaplan–Meier survival curves comparing the high and low expression of TIMM13 in SKCM by using Xiantao tool. Survival curves of OS (**A**), FPI (**B**) in the SKCM patients. OS, overall survival; PFI, progression free interval.

It was suggested that the expression level of TIMM13 mRNA in SKCM patients is higher than that in the normal control group. Therefore, whether the expression of TIMM13 was related to tumor prognosis needs further study. In this study, the expression of TIMM13 and its relationship with prognosis were detected by the Kaplan–Meier survival curve to determine whether TIMM13 can be used as a biomarker for prognosis of SKCM. It was worth noting that the high expression of TIMM13 was associated with a poorer outcome of SKCM. High expression of TIMM13 was associated with poorer OS, and PFI (Figures 3A,B).

### Correlation between immune infiltration and TIMM13 expression in SKCM

**Figure 4:**
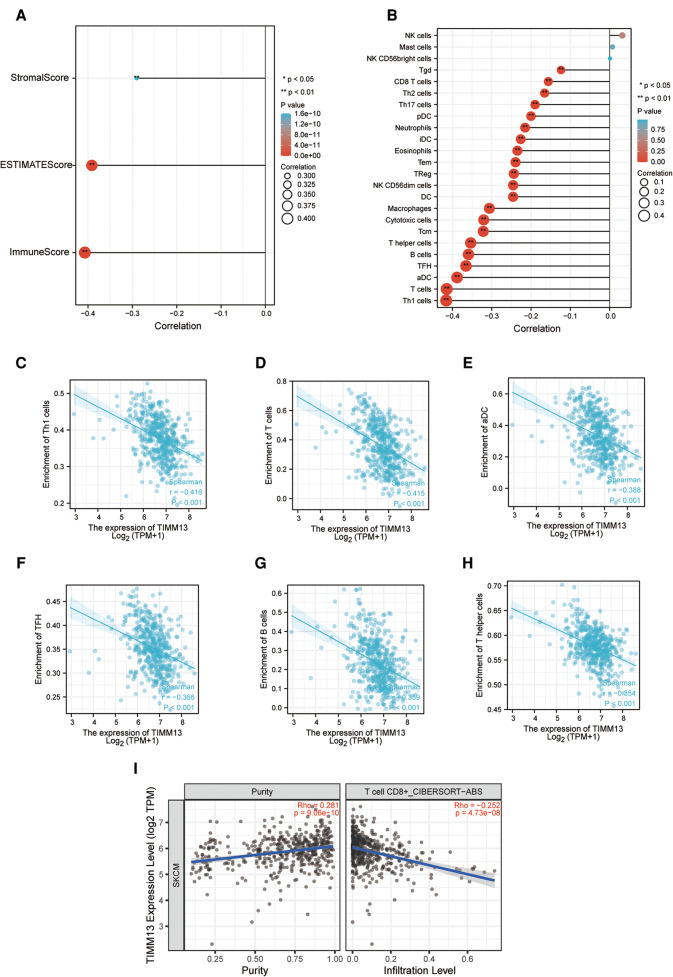
Correlation of TIMM13 expression with immune infiltration in SKCM. (**A**) Correlation of TIMM13 expression with infiltration levels of StomalScore, ESTIMATEScore, and ImmuneScore in SKCM available at Xiantao tool. (**B**) Correlation of TIMM13 expression with infiltration levels of immune cells in SKCM available at Xiantao tool. Correlation of ITGAL expression with infiltration levels of Th1 cells (**C**), T cells (**D**), aDC (**E**), TFH (**F**), B cells (**G**), and T helper in SKCM (**H**). (**I**) Correlation between the expression of TIMM13 and the abundance of TILs in SKCM validation at TIMER database.

Immune infiltration plays an important role in tumor progression. We observed whether the expression of TIMM13 affected the number of tumors infiltrating lymphocytes (TILs) in SKCM by using the Xiantao tool. As a result, the expression of TIMM13 was negatively correlated with StromalScore, ImmuneScore, and ESTIMATEScore of SKCM (rho = −0.307, *p* < 0.001; rho = −0.398, *p* < 0.001; rho = −0.393, *p* < 0.001, respectively) (Figure 4A). Our results also revealed a significant correlation between TIMM13 and TILs abundance (Figure 4B). The high expression of TIMM13 was negatively correlated with the infiltration of Th1 cells (rho = −0.416) (Figure 4C) and T lymphocytes (Rho = −0.415) (Figure 4D), aDC cells (rho = −0.338) (Figure 4E), TFH cells (rho = −0.366) (Figure 4F), B lymphocytes (Rho = −0.359) (Figure 4G) and T helper T cells (rho = −0.354) (Figure 4H). All the *p*-values above were <0.001. Similar results were obtained by detecting CD8 + T lymphocyte infiltration using the TIMER database, suggesting that TIMM13 could play a key role in inhibiting SKCM immune infiltration and was related to tumor microenvironment.

The correlation between TIMM13 and different TILs' biomarkers in SKCM was studied by using the Xiantao tool. We found that TIMM13 was associated with most TILs' markers in SKCM. Several functional T lymphocytes, including Th1, Tregs and exhausted T lymphocytes, were also investigated. In particular, TIMM13 is significantly associated with most sets of TILs immunomarkers in SKCM (Table 4).

**Table 4:** Correlation analysis between ITGAL and related genes and markers of immune cells in Xiantao tool.

Description	Gene markers	Cor	*p*
CD8^+^ T cell	CD8A	−0.373	<0.001
	CD8B	−0.353	<0.001
T cell (general)	CD3D	−0.358	<0.001
	CD3E	−0.353	<0.001
	CD2	−0.370	<0.001
B cell	CD19	−0.315	<0.001
	CD79A	−0.323	<0.001
Monocyte	CD86	−0.368	<0.001
	CD115 (CSF1R)	−0.287	<0.001
TAM	CCL2	−0.200	<0.001
	CD68	−0.165	<0.001
	IL10	−0.240	<0.001
M1 Macrophage	IRF5	−0.304	<0.001
M2 Macrophage	CD163	−0.285	<0.001
	VSIG4	−0.248	<0.001
	MS4A4A	−0.322	<0.001
Neutrophils	CD11b (ITGAM)	−0.286	<0.001
	CCR7	−0.303	<0.001
Natural killer cell	KIR2DL1	−0.131	0.004
	KIR3DL1	−0.154	<0.001
	KIR3DL2	−0.271	<0.001
	KIR2DS4	−0.133	0.004
Dendritic cell	HLA-DPB1	−0.301	<0.001
	HLA-DQB1	−0.287	<0.001
	HLA-DRA	−0.345	<0.001
	HLA-DPA1	−0.330	<0.001
	BDCA-1(CD1C)	−0.255	<0.001
	BDCA-4(NRP1)	−0.156	<0.001
	CD11c (ITGAX)	−0.2271	<0.001
Th1	T -bet (TBX21)	−0.337	<0.001
	STAT4	−0.372	<0.001
	STAT1	−0.308	<0.001
	IFN-*γ* (IFNG)	−0.365	<0.001
	TNF-α (TNF)	−0.262	<0.001
Th2	GATA3	−0.280	<0.001
	STAT5A	−0.091	0.050
Tfh	BCL6	−0.267	<0.001
	IL21	−0.384	<0.001
Th17	STAT3	−0.201	<0.001
Treg	FOXP3	−0.249	<0.001
	CCR8	−0.371	<0.001
	STAT5B	−0.217	<0.001
T cell exhaustion	PD-1 (PDCD1)	−0.288	<0.001
	PDL1(PDCD1LG2)	−0.371	<0.001
	CTLA4	−0.306	<0.001
	LAG3	−0.260	<0.001
	TIM-3 (HAVCR2)	−0.345	<0.001
	GZMB	−0.270	<0.001

Furthermore, TIMM13 was significantly associated with most of monocytes, TAM, M2 macrophages and T cell exhaustion markers in SKCM (Table 4). As shown in the table, PD-1, PDL1, CTLA4, LAG3, TIM-3, GZMB of T-cell exhaustion, and CCL-2, CD68, IL10 of TAMs, IRF5 of M1 phenotype and CD163, VSIG4 and MS4A4A of M2 phenotype were almost negatively correlated with TIMM13 in SKCM (*p* < 0.0001). It was suggested that TIMM13 could regulate exhaustion, and macrophage polarization in SKCM.

### The expression of TIMM13 is related to immunoinhibitors and immunostimulators in SKCM

**Figure 5:**
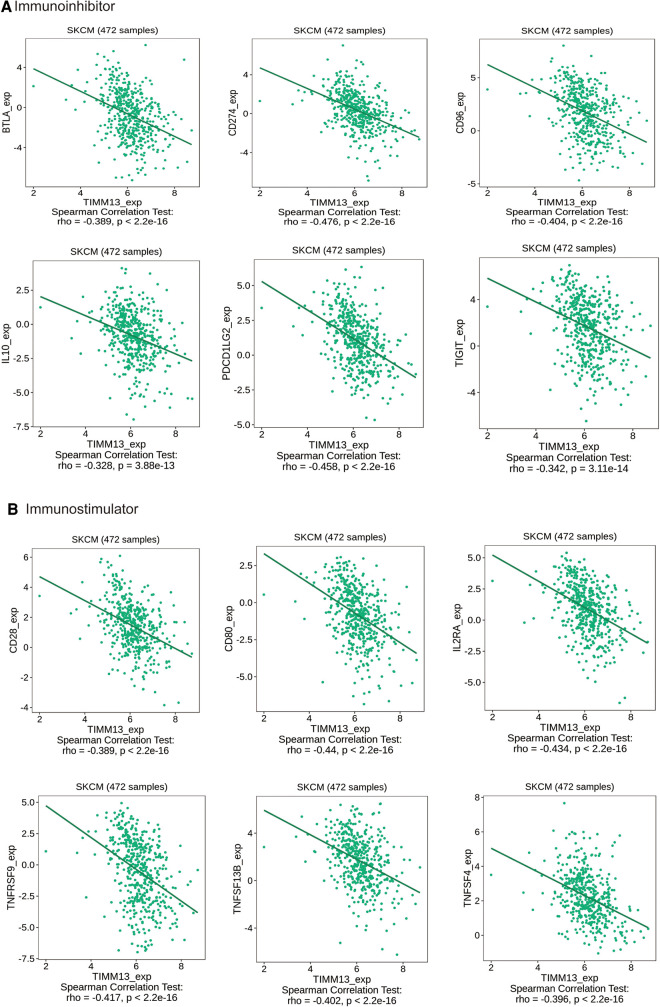
The expression of TIMM13 is associated with immunomodulators in SKCM. (**A**) Correlation between TIMM13 expression and immunoinhibitors in SKCM available at TISIDB database. (**B**) Correlation between TIMM13 expression and immunostimulators in SKCM available at TISIDB database. Color images are available online.

Immunoinhibitors and immunostimulators are substances that influence the function of the immune system. This study showed that TIMM13 was significantly associated with immunoinhibitors (*p* < 0.001), such as BTLA (rho = −0.389), CD274 (rho = −0.476), CD96 (rho = −0.404), IL10 (rho = −0.328), PDCD1LG2 (rho = −0.458), and TIGIT (rho = −0.342) (Figure 5A). The expression of TIMM13 was also significantly associated with immunostimulators (*p* < 0.001), including CD28 (rho = −0.389), CD80 (rho = −0.44), IL2RA (rho = −0.434), TNFRSF9 (rho = −0.417), TNFSF13B (rho = −0.402), TNFSF4 (rho = −0.396) (Figure 5B). These results proposed that TIMM13 was intimately engaged in the regulation of the immune interaction and might modulate tumor immune escape.

### Correlation between the TIMM13 expression and chemokines/receptors in SKCM

**Figure 6:**
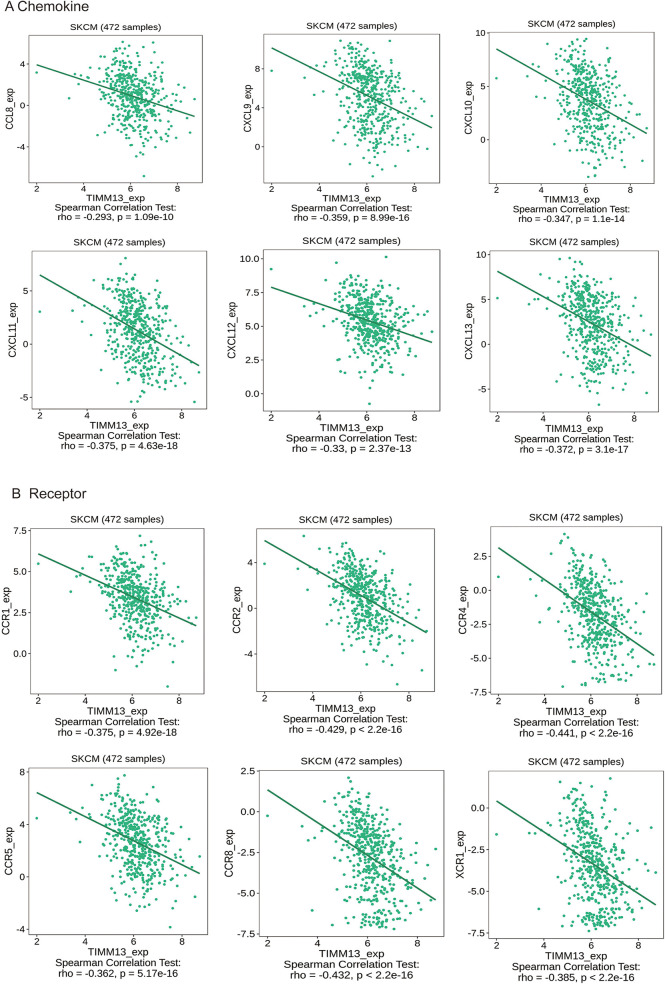
Correlation between the expression of TIMM13 and chemokines in SKCM. (**A**) Correlation between TIMM13 expression and chemokines in SKCM available at TISIDB database. (**B**) Correlation between TIMM13 expression and chemokine receptors in SKCM available at TISIDB database. Color images are available online.

Chemokines played a great function in controlling the infiltration degree of immune cell. This research implicated a significant association between TIMM13 expression and chemokines. For instance, the expression of TIMM13 was significantly associated with CCL8 (rho = −0.293), CXCL9 (rho = −0.359), CXCL10 (rho = −0.347), CXCL10 (rho = −0.347), CXCL11 (rho = −0.375), CXCL12 (rho = −0.33), and CXCL13 (rho = −0.372) (Figure 6A). All the values of *p* were < 0.001. At the same time, we found that the expression of TIMM13 was also significantly related with chemokine receptors (*p* < 0.001), including CCR1 (rho = −0.375), CCR2 (rho = −0.429), CCR4 (rho = −0.441), CCR5 (rho = −0.362), CCR8 (rho = −0.432), XCR1 (rho = −0.385) (Figure 6B). These results further suggested that TIMM13 may be an immunoregulatory factor in SKCM.

### Construction and validation of a nomogram

**Figure 7:**
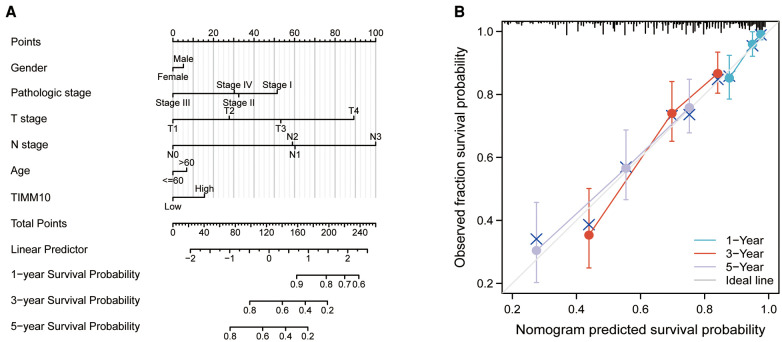
Nomogram model construction. (**A**) Nomogram. (**B**) Calibration plot.

A nomogram was built for the training set based on the gender, pathologic stage, TNM stage, age, and TIMM13 expression (Figure 7A). The C-index of the nomogram was 0.715(0.693–0.736). The calibration plots for the 1-, 3-, and 5-year survivals indicated good agreement between the actual observations and the predictions (Figure 7B). These results indicated that the prediction performance of the nomogram was good.

### Histologic analysis

**Figure 8:**
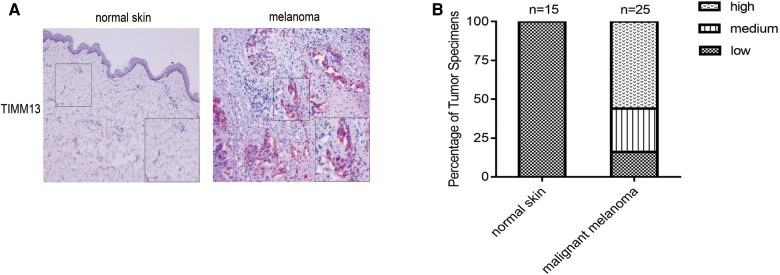
Gene expression of TIMM13 in melanoma tissue and normal skin specimens. Using IHC staining, TIMM13 was expressed at higher levels in the melanoma tissue (*n* = 25) when compared to normal skin (*n* = 15). (**A**) IHC staining. (**B**) Quantification of the TIMM13 levels. IHC: Immunohistochemistry. IHC stain, AEC, original magnification: 100× (inset, IHC stain, AEC, original magnification: 400×).

We next explored the protein expression levels of TIMM13 between melanoma and normal skin tissues. The IHC staining results showed that TIMM13 showed higher expression levels in melanoma tissues (*n* = 25) than in normal skin tissues (*n* = 15) (Figures 8A,B). The patients' characteristics were shown in Table 5.

**Table 5:** Clinical characteristics of melanoma patients.

ID	Sex	Age,y	Location	Primary/metastasis
Case1	M	54	Right plantar	Primary
Case2	F	35	Left plantar	Primary
Case3	M	82	Left plantar	Primary
Case4	F	39	Right ankle	Primary
Case5	M	62	Left plantar	Metastasis
Case6	M	44	Left plantar	Metastasis
Case7	M	55	Right plantar	Metastasis
Case8	F	57	Right plantar	Metastasis
Case9	F	48	Face	Primary
Case10	M	73	Left plantar	Primary
Case11	M	64	Left plantar	Metastasis
Case12	M	65	Right plantar	Primary
Case13	M	66	Right plantar	Metastasis
Case14	F	58	Right plantar	Primary
Case15	F	64	Right plantar	Primary
Case16	F	74	Right arm	Primary
Case17	M	66	Right plantar	Metastasis
Case18	M	57	Right plantar	Primary
Case19	M	52	Back	Primary
Case20	M	63	Left plantar	Metastasis
Case21	F	74	Right plantar	Metastasis
Case22	F	71	Left ankle	Primary
Case23	M	63	Left plantar	Primary
Case24	M	41	Left arm	Metastasis
Case25	M	52	Head	Metastasis

F, female; M, male.

## Discussion

In this study, the expression level of TIMM13 in SKCM and its clinical significance were comprehensively studied by bioinformatics. Our results showed that poor prognosis was in accordance with high expression of TIMM13 in SKCM. The expression of TIMM13 is closely related to the infiltration of various immune cells, immunomodulators, chemokines and receptors in SKCM. Therefore, our study provided new insights into the key functions of TIMM13, which might be a prognostic biomarker related to immune infiltration of SKCM.

TIMM13 partners with TIMM8a in the mitochondrial intermembrane space to form a 70 kDa complex and facilitates the import of the inner membrane substrate TIMM23 (9). Kim SH et al. reported that TIMM13 functions as a target gene of miRNA-1273g-3p to contribute to Alzheimer's Disease pathogenesis by regulating expression of mitochondrial genes (22). Roesch K et al. reported that human deafness dystonia syndrome was caused by a defect in assembly of the DDP1/TIMM8a-TIMM13 complex. Timm13 is related to Mohr-Tranebjaerg Syndrome and Visual Cortex Disease. Among its related pathways are Peroxisomal lipid metabolism. Several studies have shown that TIMM13 was differently expressed in metastatic susceptibility (13), hepatocellular carcinoma (14), breast cancer (15) et al. These researchers suggested that TIMM13 might have a significant influence on cancer, and may be a novel target in treating a variety of malignancies. However, the possible function of TIMM13 in regulating tumor immunity and its clinical significance in SKCM are still unknown.

Therefore, we computed the TIMM13 expression for SKCM using the Xiantao tool and the UALCAN database. We found that TIMM13 was abnormally expressed in the tissues of various malignant tumors. TIMM13 in SKCM was significantly higher than that in adjacent tissues. Using the HPA database, we also found that TIMM13mRNA and protein levels in most SKCM samples were higher than those in matched paracancer samples. It was suggested that the expression level of TIMM13 could be used as a diagnostic indicator of SKCM with the AUC is 0.757. In addition, in order to determine whether TIMM13 could be used as a biomarker for prognosis, we analyzed the correlation between TIMM13 expression and OS, and FPI in SKCM patients with the Xiantao tool. Furthermore, to confirm whether TIMM13 can be used as a prognostic biomarker, we used the xiantao tool to analyze the correlation between the TIMM13 expression and OS, DSS, and FPI in SKCM patients. Notably, analysis of this database indicated that the higher TIMM13 expression correlated with HR for worse OS DSS, and poorer PPS of SKCM. In addition, upregulated TIMM13 expression had a significant correlation with a worse prognosis of SKCM in T4 stage, N1 stage, N2 stage, N3 stage. Taken together, these observations supported our hypothesis that TIMM13 was a prognostic biomarker in SKCM.

Additionally, this study found that TIMM13 was correlated with the degree of immune infiltration of SKCM. In the cancer microenvironment, immune cell infiltration had been proved to play a key role in the development and progression of cancer (23, 24). TIMM13 is a chaperone protein, which plays a key role in transporting cytoplasmic proteins into the mitochondrial inner membrane. At present, it is not clear whether the expression of TIMM13 is related to the immune infiltration of SKCM. Therefore, we systematically discussed the relationship between TIMM13 expression and immune infiltration degree of SKCM. Based on our results, it was found for the first time that TIMM13 modulates immune infiltration through SKCM.

The expression of TIMM13 was closely related to the expression of TILs in CD8 + T cells, CD4 + T cells, Treg cells, and TAM cells. Meanwhile, the increase of TIMM13 expression was related to immunoregulators, chemokines and receptors. Additionally, the study proposed the relationship between TIMM13 expression and TILs’ marker genes of SKCM. The expression of TIMM13 was associated with M2 macrophage markers CD163, VSIG4, and MS4A4A, while M1 macrophage markers NOS2 and IRF5 were slightly associated with the expression of TIMM13. Macrophages played an important role in tumor proliferation (25), angiogenesis (26), invasion (27), metastasis (27) and tumor immunity (28). These results demonstrated that TIMM13 had the potential function of regulating TAMs polarization. The up-regulation of TIMM13 was closely related to Tregs markers (FOXP3, CCR8) and T lymphocyte depletion markers (PD1, CTLA4, LAG3). Blocking immune checkpoints is the main strategy of immunotherapy (29). The most important thing is to enhance the response of tumor cells to immune checkpoint inhibitors and cytokines (30, 31). According to TISIDB, TIMER and Xiantao tool, our results showed that the up-regulation of TIMM13 expression was not only related to PD1 and CTLA4, but also significantly related to cell response to chemokines. These results suggested that targeting TIMM13 may be a strategy to improve the efficacy of immunotherapy. It was also suggested that TIMM13 played an important role in the recruitment and regulation of TILs in SKCM, and its molecular mechanism and role in the regulation of tumor microenvironment deserve further study.

However, there are also some limitations in our research. One limitation is that most of the data is based on online platforms, which are constantly updated and extended; So, the research results may be influenced. Secondly, our study did not include any information about complications and therapy options. Thirdly, *in vivo* and *in vitro* experiments are not to verify the role of TIMM13 in SKCM. However, in future research, we are committed to paying more attention to the background information of patients and conducting experiments to further verify the expected results.

## Conclusions

The increased expression of TIMM13 was closely related to poor prognosis of SKCM and increased immune infiltration of CD8 + T lymphocytes, C4 + T lymphocytes, and myeloid dendritic cells. The expression of TIMM13 might be involved in the regulation of M2, Treg, and T -cell exhaustion. So, this study suggested that TIMM13 might have a new potential function in the regulation of immune cell infiltration in SKCM patients.

## Data Availability

The original contributions presented in the study are included in the article/Suplementary Material, further inquiries can be directed to the corresponding author/s.
